# Successful therapy of complex regional pain syndrome after hip arthroscopy for femoroacetabular impingement syndrome: a case report

**DOI:** 10.1186/s13256-023-04276-3

**Published:** 2024-01-02

**Authors:** V. Twardy, R. von Eisenhart-Rothe, I. J. Banke

**Affiliations:** Klinik und Poliklinik für Orthopädie und SportorthopädieKlinikum rechts der Isar, TU München, Ismaninger Str. 22, 81675 München, Deutschland

**Keywords:** Hip arthroscopy, Femoroacetabular impingement syndrome (FAIS), Complex regional pain syndrome (CRPS), Arthroscopic complications, Case report

## Abstract

**Background:**

Complex regional pain syndrome is a neuropathic pain disorder associated with ongoing pain that persists beyond the usual expected tissue healing time and that is disproportionate to the degree of tissue injury present. Complex regional pain syndrome after hip arthroscopy has not been reported before. Hip arthroscopy is a fast-growing domain that could lead to an increasing number of complex regional pain syndrome cases, probably owing to the high traction forces that are necessary.

**Case presentation:**

We report the case of a 30-year-old German female semiprofessional tennis player who presented with complex regional pain syndrome type I in the lower leg 3 weeks after hip arthroscopy for femoroacetabular impingement syndrome with suture anchor labral repair and femoroplasty. After 2 months of immediate multimodal conservative therapy including administration of gabapentin, prompt full weight-bearing, and intensified physiotherapy, complete recovery was achieved.

**Conclusion:**

Complex regional pain syndrome does occur after elective hip arthroscopy. Disproportionate postoperative pain or other symptoms raising suspicion of complex regional pain syndrome should be promptly evaluated and treated through a multimodal approach. Postless hip arthroscopy may be advantageous.

## Background

Complex regional pain syndrome (CRPS) is a neuropathic pain disorder associated with ongoing pain that persists beyond the usual expected tissue healing time and that is disproportionate to the degree of tissue injury present [1].

It is accompanied by abnormalities including allodynia, hyperalgesia, sudomotor and vasomotor abnormalities, and trophic changes [1]. CRPS often occurs after surgery or trauma [1–3]. There are two subtypes: type I (reflex sympathetic dystrophy) and type II (causalgia) after nerve trauma [1, 2]. Diagnosis is clinical and based on the Budapest criteria [1]. Therapy should be multimodal, including medication, physical therapy, and nerve stimulation if needed. Hip arthroscopy is a soft tissue-saving standard procedure for femoroacetabular impingement syndrome (FAIS). The aim of this case report is to present a unique case of CRPS after hip arthroscopy.

## Case report

**Fig. 1:**
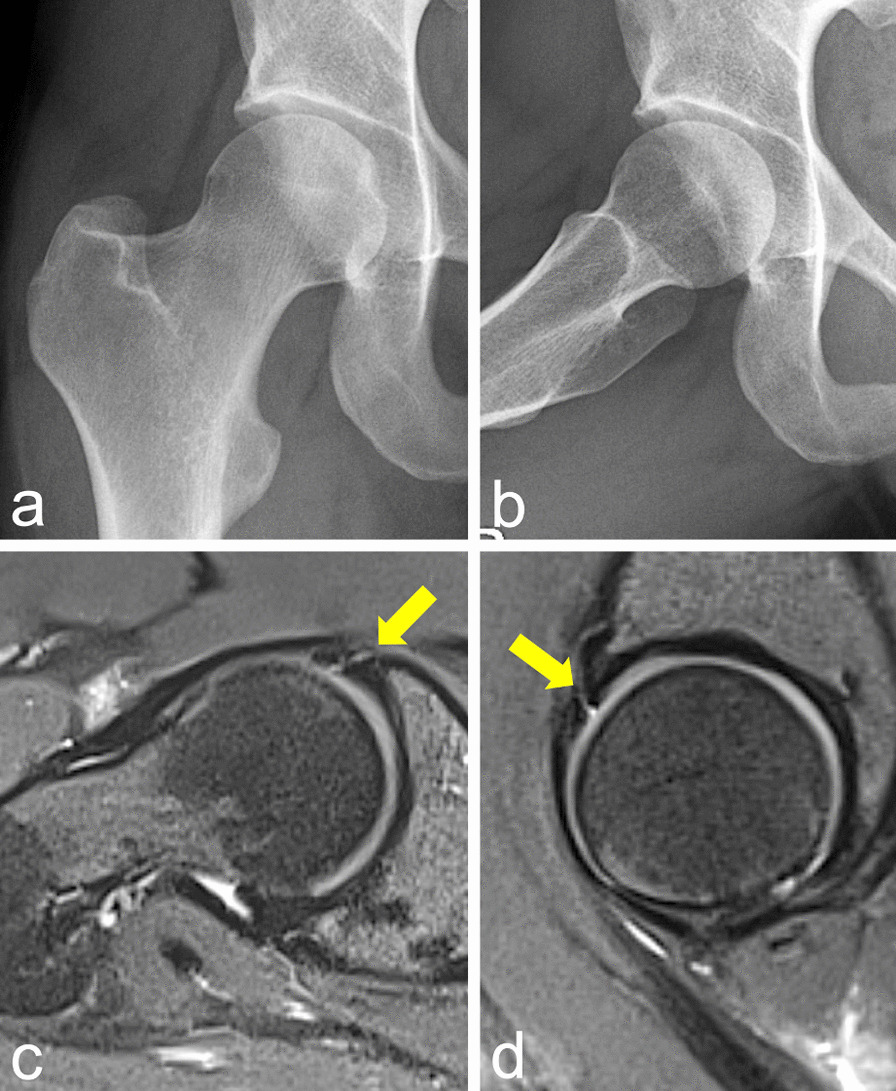
**a**–**d** Preoperative imaging of right hip. **a**, **b** Pelvis anteroposterior radiograph and frog-lateral view with mild cam deformity and borderline pincer deformity. **c**, **d** High-resolution axial and sagittal magnetic resonance imaging with labral tear at the anterolateral acetabulum (arrow)

A 30-year-old German female semi-professional tennis player presented with immobilizing groin pain during sporting activities. Clinical examination (impingement and labral tests), radiography, and high-resolution magnetic resonance imaging (MRI) of the right hip revealed a FAIS of cam type accompanied by a labral tear (Fig. 1a–d). After positive intraarticular infiltration test, the patient underwent hip arthroscopy by a highly experienced hip surgeon at a nationwide reference center.

**Fig. 2:**
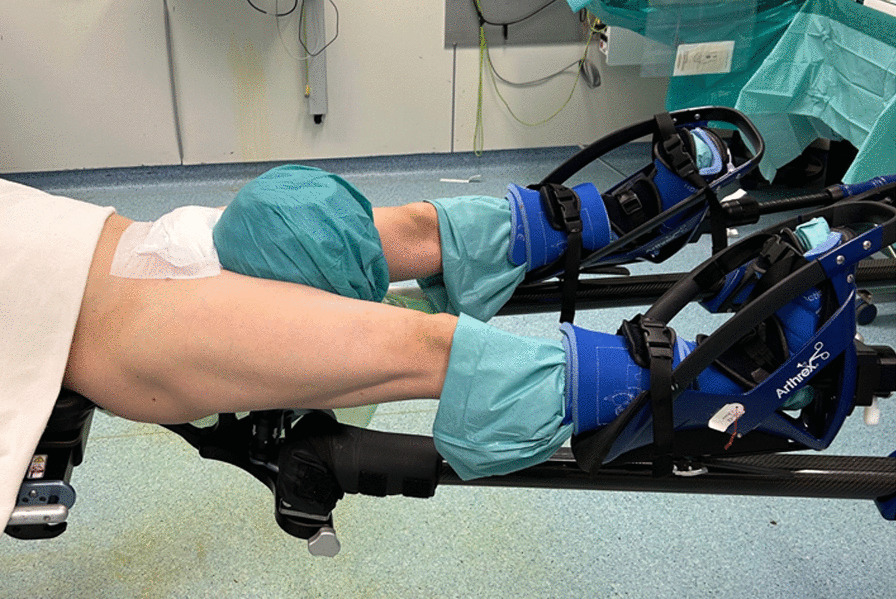
Patient positioning for supine hip arthroscopy in hip distraction system with 30-cm foam padded perineal post and well-padded traction boots

**Fig. 3:**
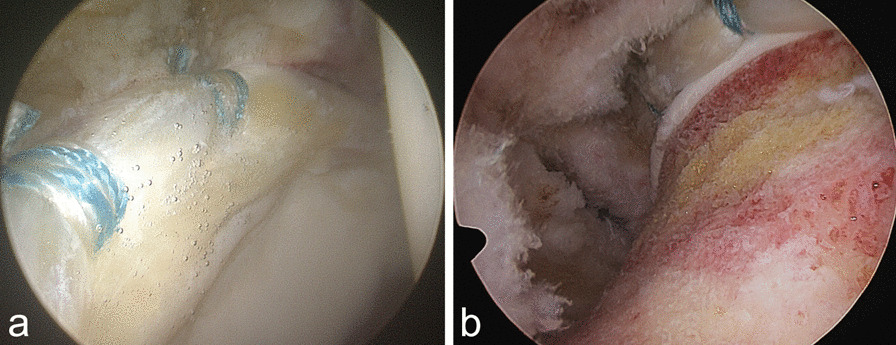
**a**, **b** Arthroscopic final view. **a** Central compartment. Labrum repair with two suture anchors, underneath acetabular chondral carpet delamination. **b** Peripheral compartment with osteochondroplasty of the femoral head–neck junction, labrum repair, and capsule closure after capsulotomy

The patient was positioned supine in a standard hip distraction system (Arthrex^®^) with 30-cm foam padded perineal post and well-padded traction boots (Fig. 2). Regional anesthesia block was not carried out, according to our standard. Hip arthroscopic labral repair with two suture anchors was performed (Fig. 3a, b). Specific cartilage therapy or pincer correction was not indicated. Once central compartment work was completed, traction was released after 30 min. Cam resection was done in the peripheral compartment (30 min). After skin closure, periportal local anesthesia was applied.

**Fig. 4:**
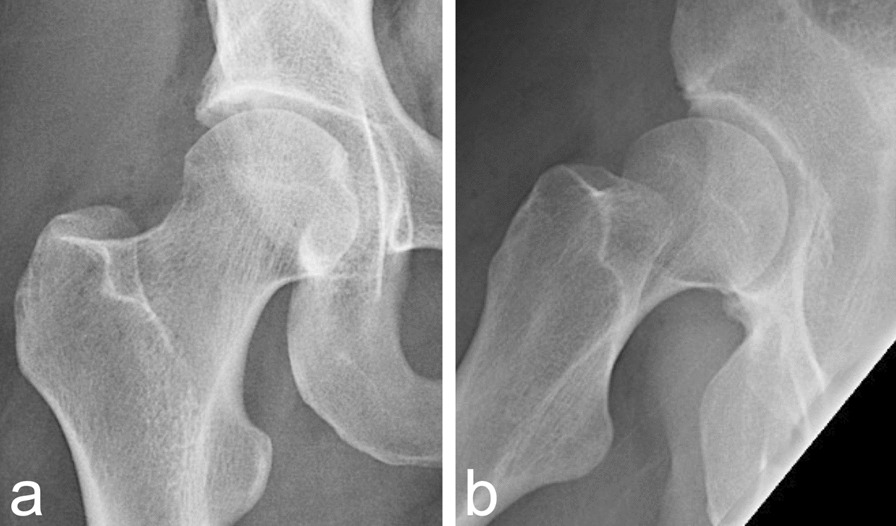
**a, b** Postoperative imaging of right hip (**a**, **b**). Pelvis anteroposterior radiograph and frog-lateral view with spherical femoroplasty

Postoperative X-rays showed a correct spherical femoroplasty (Fig. 4a, b). Physiotherapy started immediately, including continuous passive motion. Pain level was normal during hospitalization, with discharge at the third postoperative day.

Physiotherapy (three times/week), lymphatic drainage, and partial weight-bearing (15 kg) were performed correctly. Pain medication, ossification (celecoxib 100 mg 1–0–1), and venous thromboembolism prophylaxis were taken daily.

**Fig. 5:**
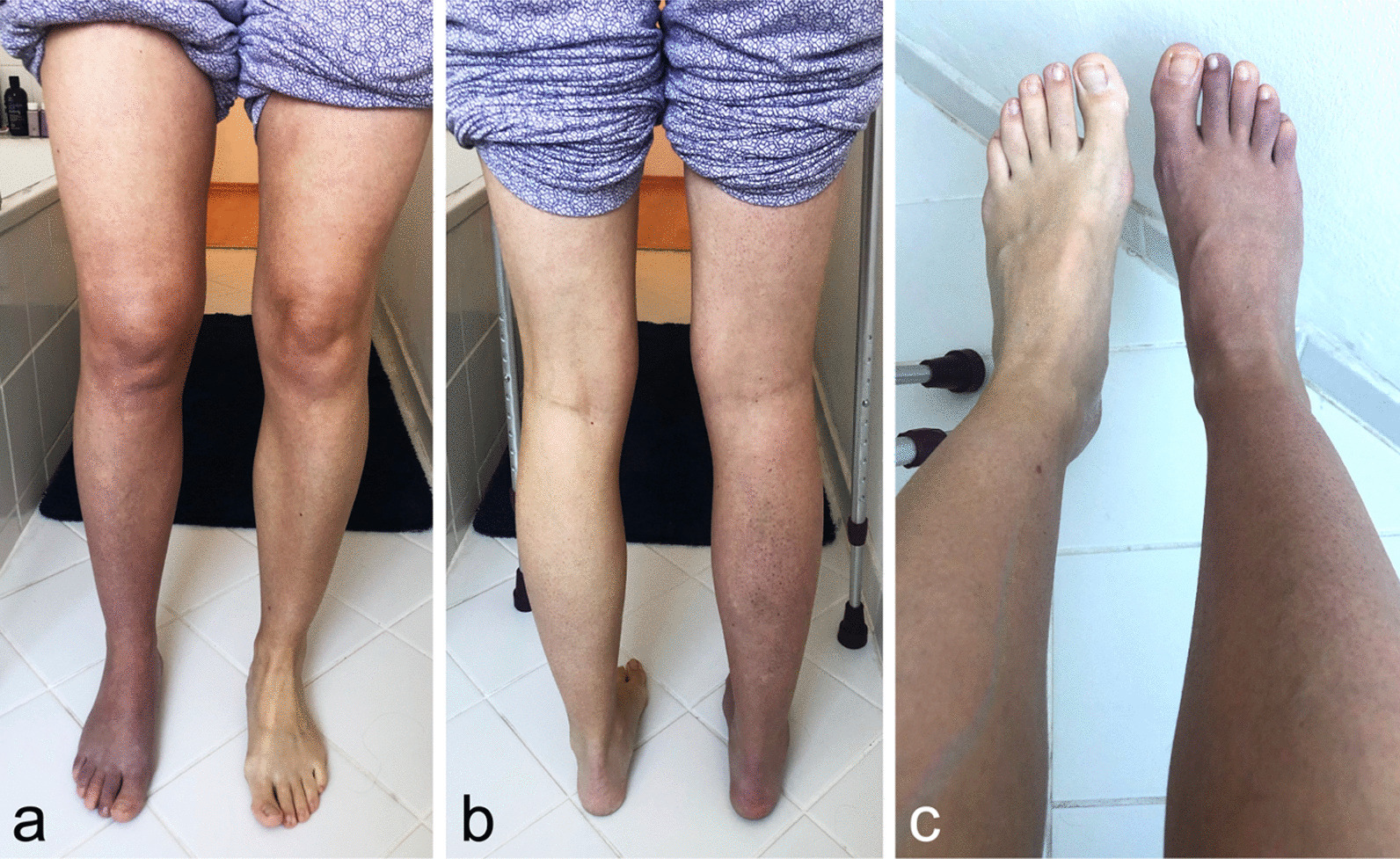
**a**–**c** 3 weeks postoperatively: Right lower leg shows impressive bluish discoloration with generalized swelling of the right foot

Three weeks postoperatively, the patient presented with a cold, blue, swollen, and painful lower right leg (VAS 7.5/10) (Fig. 5a–c). Neurological examination revealed mild paresis of foot extensor and flexor (4/5), brisk muscle reflexes, a temperature difference, and paresthesia in the toes. Deep vein thrombosis and arterial insufficiency were excluded by vascular surgeon. Blood test was inconspicuous. No previous illnesses are known. The patient was concerned that she could not do sports again.

In conclusion, the diagnosis of a fulminant CRPS type I was confirmed by two independent neurologic and one anesthetic specialists. Fortunately, the interdisciplinary cooperation was uncomplicated. Immediately, therapy was accommodated and intensified: Daily physiotherapy with free range of motion and return to full weight-bearing. Multimodal pain management including nonsteroidal antiinflammatory drugs (NSAIDs) (ibuprofen, 400 mg 1–1–1) and neuropathic pain medication (gabapentin: week 1–2, 300 mg three times a day, week 3–4, 200 mg three times a day) was performed. The patient was compliant.

**Fig. 6:**
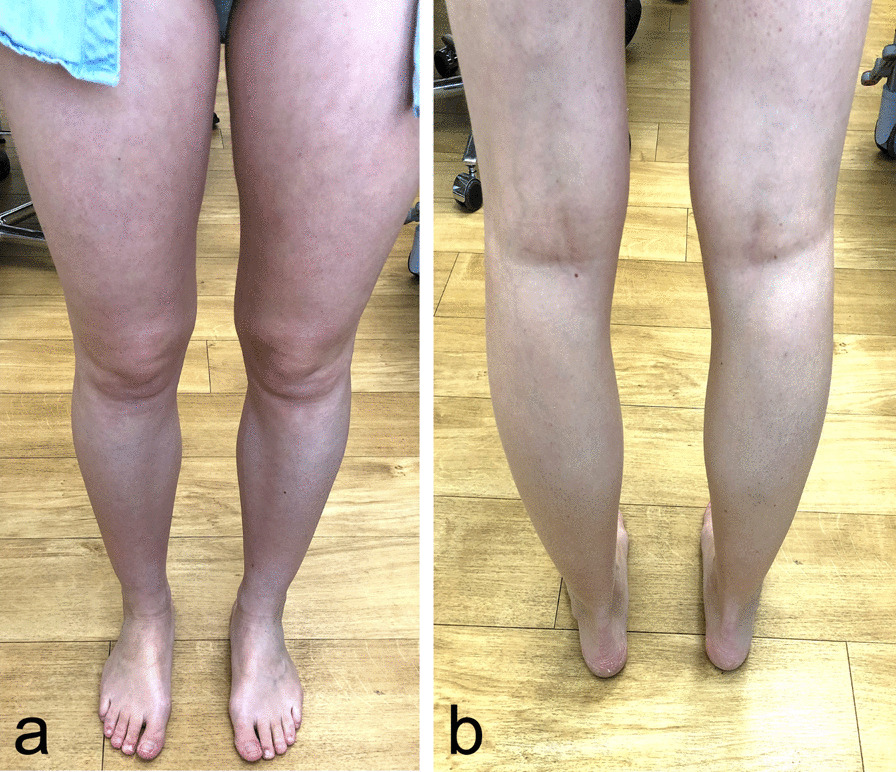
**a**, **b** 12 months postoperatively: Complete recovery with lack of complex regional pain syndrome

**Fig. 7:**
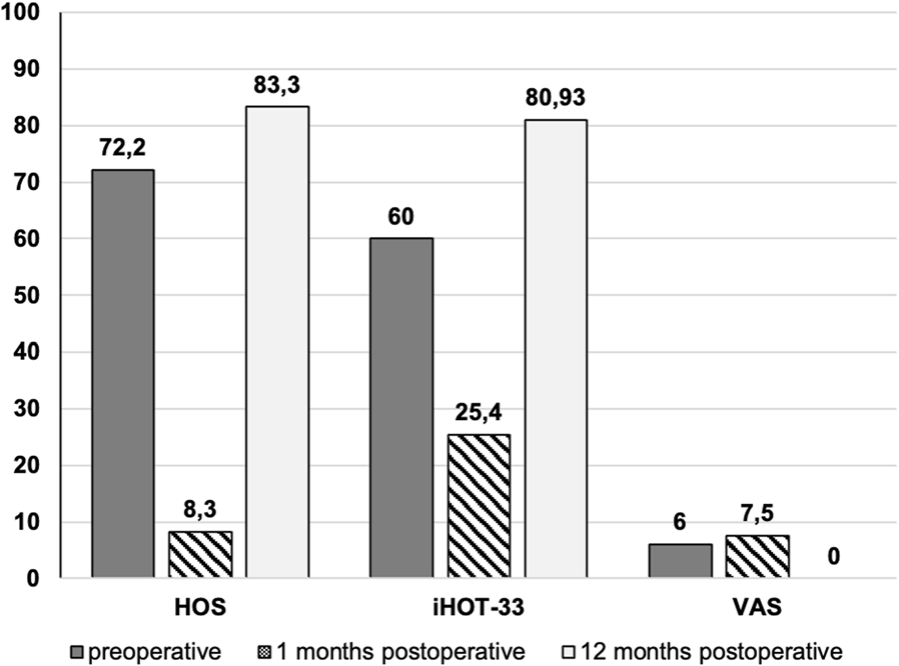
Clinical outcome scores preoperatively, at complex regional pain syndrome maximum (1 month postoperative), and 12 months postoperatively. Hip Outcome Score (HOS), 33-item International Hip Outcome Tool (iHOT-33), and visual analog scale (VAS) for pain

**Fig. 8:**
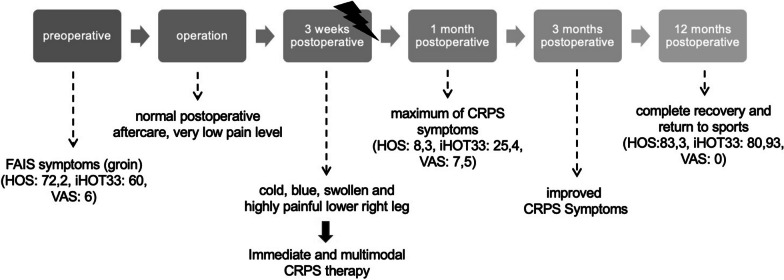
Timeline: historical and current information from episode of care

In the clinical and neurological follow-up at 3, 6, and 9 months later, the patient’s CRPS symptoms improved constantly (Fig. 6a, b). Clinical outcome scores were obtained preoperatively—1 month (CRPS maximum) postoperatively—12 months postoperatively via Hip Outcome Score (HOS: 72.2–8.3–83.3), 33-item International Hip Outcome Tool (iHOT-33: 60–25.4–80.93), and visual analog scale for pain (VAS: 6–7.5–0) (Fig. 7). After 12 months, a delayed (compared with our normal FAIS outcome) but finally full return to sports including competition level (tennis) could be achieved with high patient satisfaction until last check-up 19 months postoperatively (Fig. 8).

## Discussion

CRPS is an uncommon but debilitating postoperative complication that can negatively impact patient satisfaction and quality of life [4]. To our knowledge, no cases of CRPS after hip arthroscopy and/or FAIS therapy have been reported before. To avoid irreversible damage, it is important to determine the diagnosis early and to identify risk factors (female gender, fibromyalgia, rheumatoid arthritis, neuropathic inflammation, autonomic nervous system alterations, and psychological factors) [3, 5]. Kumar *et al.* reported two cases of CRPS type I after open trauma hip surgery [6]. Commonalities with our case are gender (female), young age, and early start of symptoms after surgery. It was recommended to commence weight bearing at the earliest time possible [6]. Also, in our case, full weight bearing had a positive effect. Moretti *et al.* suggested a higher prevalence of CRPS type 1 in younger people and in lower limbs than in general population, but confirmed a higher prevalence in females [9]. They also described some cases of CRPS type I in athletes [7]. Although hip arthroscopy is less invasive than open hip surgery, it is important to mention the force needed on the leg during distraction with this method (Fig. 2). This may lead to a circulatory disorder and could trigger CRPS. Therefore, it is important to reduce the traction force as much as possible. The future use of postless hip arthroscopy may be advantageous for CRPS prevention.

Interdisciplinary management plays a key role in treatment of CRPS [4]. As described above, early diagnosis and therapy with full weight bearing and multimodal pain management including neuropathic medication with gabapentin are the key points to achieve full recovery, as in our case. Some authors suggest pre- or postoperative vitamin C supplementation and sympathetic block to treat CRPS [8, 9].

## Conclusion

CRPS does occur after elective hip arthroscopy. Disproportionate postoperative pain or other symptoms raising suspicion of CRPS should be promptly evaluated and treated through a multimodal approach to prevent irreversible damage.

## Data Availability

The datasets used and/or analyzed during the current study are available from the corresponding author on reasonable request.

## Abbreviations

AP: Anteroposterior
CRPS: Complex regional pain syndrome
FAIS: Femoroacetabular impingement syndrome
HOS: Hip Outcome Score
iHOT-33: 33-Item International Hip Outcome Tool
NSAIDs: Nonsteroidal antiinflammatory drugs
VAS: Visual analog scale
